# Hydrothermal Synthesis of Hierarchical SnO_2_ Nanostructures for Improved Formaldehyde Gas Sensing

**DOI:** 10.3390/nano12020228

**Published:** 2022-01-11

**Authors:** Pengyu Ren, Lingling Qi, Kairui You, Qingwei Shi

**Affiliations:** 1 College of Architecture and Urban Planning, Chongqing Jiaotong University, Chongqing 400074, China; pengyu_ren@cqu.edu.cn; 2 School of Management Science and Real Estate, Chongqing University, Chongqing 400044, China; shiqw@cqu.edu.cn; 3 Business College, Southwest University, Chongqing 402460, China

**Keywords:** indoor air, sensor, synthesis, tin oxide, hierarchical structure

## Abstract

The indoor environment of buildings affects people’s daily life. Indoor harmful gases include volatile organic gas and greenhouse gas. Therefore, the detection of harmful gas by gas sensors is a key method for developing green buildings. The reasonable design of SnO_2_-sensing materials with excellent structures is an ideal choice for gas sensors. In this study, three types of hierarchical SnO_2_ microspheres assembled with one-dimensional nanorods, including urchin-like microspheres (SN-1), flower-like microspheres (SN-2), and hydrangea-like microspheres (SN-3), are prepared by a simple hydrothermal method and further applied as gas-sensing materials for an indoor formaldehyde (HCHO) gas-sensing test. The SN-1 sample-based gas sensor demonstrates improved HCHO gas-sensing performance, especially demonstrating greater sensor responses and faster response/recovery speeds than SN-2- and SN-3-based gas sensors. The improved HCHO gas-sensing properties could be mainly attributed to the structural difference of smaller nanorods. These results further indicate the uniqueness of the structure of the SN-1 sample and its suitability as HCHO- sensing material.

## 1. Introduction

Indoor environments are very important to people’s livelihoods. Monitoring indoor ambient gas is a key method for developing green buildings. In our daily life, formaldehyde (HCHO) is deemed as one of the most common and serious indoor air pollutants [1,2,3]. It has been considered to be a major threat because it can be easily emitted from newly decorated buildings, causing irritating reactions in the eyes, nose, and throat and resulting in coughing and even serious diseases [4,5,6]. Consequently, monitoring HCHO in a specific environment is particularly pressing for people’s safety and health [7,8].

In fact, chemical gas sensors based on semiconducting oxide materials play an important role in monitoring toxic volatile organic compounds’ vapor because of low cost and good gas-sensing properties [9,10]. However, microstructures and the surface area of semiconducting oxides closely affect their actual gas-sensing properties [11,12]. Fortunately, these vital factors could be tailored via rationally designed architectures [13]. SnO_2_ is a kind of semiconducting oxide with a wide bandgap, which has been widely researched as a gas-sensing material. Gas sensors based on SnO_2_ nanostructures have advantages of good chemical and thermal stability as well as high electron mobility. SnO_2_ becomes the most practical sensing material because of its excellent inherent characteristics of oxygen exchange with the atmosphere [14]. Pan et al. synthesized metal-organic framework-derived porous SnO_2_ nanosheets and explained its excellent formaldehyde gas-sensing abilities [15]. Zhang et al. prepared porous core-shell SnO_2_ spheres and found that core-shell spheres showed enhanced VOC sensing compared to the SnO_2_ particles [16]. In particular, construction of unique hierarchical architectures assembled by low dimensional nanostructures is significant in enhancing gas-sensing performance. For instance, Liu et al. prepared five types of SnO_2_ nanostructures by using a simple hydrothermal method and compared their methanol gas-sensing performances [17]. The results showed that hierarchical SnO_2_ nanoflowers displayed the highest gas-sensing behaviors. Although much progress about SnO_2_ sensing materials has been achieved, to the best of our knowledge, controllable synthesis of hierarchical gas-sensing materials assembled by low dimensional nanostructures is still a major challenge.

Hydrothermal synthesis is a common technique for preparing nanomaterials. It has many advantages, such as easy processing, high yield, better control of structure growth, and high crystallinity [18]. Hence, in this study, three kinds of hierarchical SnO_2_ nanostructures based on one-dimensional nanorods were successfully synthesized via controlling hydrothermal time and amount of citric acid. The microstructures and surface morphologies of the as-synthesized SnO_2_ spherical architectures were analyzed by XRD and SEM techniques. Three hierarchical SnO_2_ architectures with different basic units were utilized as gas-sensing film in studying gas-sensing properties. Working temperature, sensor response, and HCHO gas concentrations were comparatively investigated. The sensor response and response and recovery speeds were tested to determine whether unique rod-sphere structures could affect gas-sensing performance. The results indicate that the SN-1 sample-based gas sensor displayed the highest response value (53.6) and fastest response and recovery speed (5/9 s) at 275 °C towards 50 ppm formaldehyde. In addition, a possible gas-sensing mechanism was also discussed. 

## 2. Materials and Methods

### 2.1. Chemical Preparation

For hierarchical SnO_2_ architectures, the synthesis process is as follows. It is noted that all the chemical reagents have analytical purity and are used directly. Firstly, a mixed solution was obtained using 20 mL deionized water and 20 mL ethanol by stirring. Then, 0.8 g of NaOH and 0.9 g of SnCl_4_•5H_2_O were subsequently dissolved into the above-mentioned mixed solution by magnetic stirring for 30 min. After that, 0.04 g of nitric acid was added into the obtained solution. After stirring for 30 min, the solution was loaded into a 50 mL Teflon-lined stainless-steel autoclave and maintained at 160 °C for 24 h. When the hydrothermal reaction was completed, the autoclave was cooled to room temperature naturally. The precipitates in the autoclave were collected by applying centrifugation and washed using deionized water and ethanol three times to remove impurities and ions on the product’s surface. Finally, washed precipitates were dried at 60 °C for 24 h. As a comparison, another sample was prepared by reducing hydrothermal time to 12 h, and other steps were not altered. The samples after 24 h and 12 h reaction were denoted as SN-1 and SN-2, respectively. In addition, a sample was synthesized by increasing the amount of citric acid to 0.08 g, and other steps were the same as SN-2. The product was recorded as SN-3.

### 2.2. Characterization

The crystal structure and purity of SnO_2_ nanostructures were characterized by using X-ray powder diffraction (XRD, Rigaku D/Max-1200, Rigaku Corporation, Tokyo, Japan). Structural and morphological features of the three SnO_2_ powders were analyzed by employing field emission scanning electron microscopy (FE-SEM and JSM-7800F, JEOL Corporation, Tokyo, Japan).

### 2.3. Gas Sensor Fabrication and Measurement

The detailed steps of gas sensor fabrication are as follows. Firstly, a well-distributed paste was formed by mixing as-prepared SnO_2_ powders with a mixture of deionized water and polyethylene glycol in an agate mortar. The formed slurry was coated onto a commercial device with a fine brush. The thickness of sensing film was about 100 μm estimated by using optical microscopy. The device contains a ceramic substrate and two Au electrodes attached with four Pt wires. Then, a tiny Ni-Cr ally wire was inserted into the ceramic substrate to serve as the heating source. The fabricated gas sensors based on the as-prepared SnO_2_ powder were aged at 200 °C in order to improve their stability. Finally, gas- sensing tests were carried out by an intelligent gas-sensing test system (CGS-8) [19,20]. Two fans were equipped in the test chamber with a volume of 18 L. The gas concentration was controlled by a gas-mixing system (RSC2000-A, Elitetech Co., Beijing, China). In order to obtain a certain gas concentration, a given amount of commercially available target gas was injected by a needle and evaporated into the required concentration in the chamber. The operating temperatures of the gas sensor were set in the range of 125 to 375 °C. The surface interaction of the gas sensor results in conductivity changes and resistance changes. Sensor resistance values in the air (denoted as R_a_) and in the target gas (denoted as R_g_) were continuously recorded by the built-in test system with a time interval of 1 s, and gas sensor responses (R) were calculated as the ratio of R_a_ and R_g_ (R = R_a_/R_g_) for reducing formaldehyde gas. Response times comprise the time required for the gas sensor to achieve 90% of final resistance value in the target gas’ atmosphere, and recovery times were the times needed for the gas sensor to reach to 90% of baseline resistance value after being retrieved from the target gas. The error rate is less than 2% in the measurement.

## 3. Results and Discussion

### 3.1. Sample Characterization

**Figure 1:**
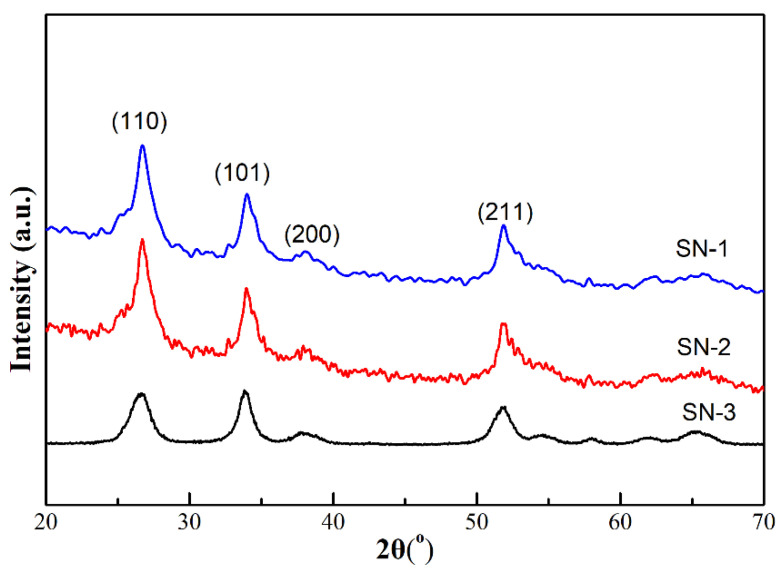
XRD patterns of as-prepared SnO_2_ samples (blue: SN-1 sample; red: SN-2 sample; black: SN-3 sample).

XRD patterns, shown in Figure 1, exhibit purity and crystal structures of three SnO_2_ samples (SN-1, SN-2 and SN-3). Evidently, all diffraction peaks are matched to the tetragonal rutile SnO_2_ phase (JCPDS No. 41-1445) with lattice constants of a = b = 4.7382 $\overset{o}{A}$ and c = 3.1871 $\overset{o}{A}$. In addition, no other diffraction peaks from impurities were found. It is undoubtable that SnO_2_ samples with high purities have been prepared by the hydrothermal method. Moreover, the crystallite size of these SnO_2_ samples could be calculated by using the Debye–Scherrer formula [21]. The average size for SN-1, SN-2, and SN-3 is 16, 16, and 13 nm, respectively.

**Figure 2:**
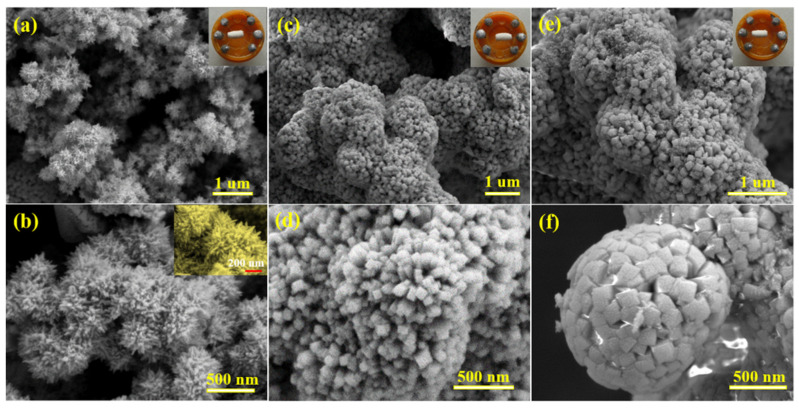
FE-SEM images of three SnO_2_ samples: (**a**,**b**) SN-1, (**c**,**d**) SN-2, (**e**,**f**) SN-3 (the inserts of (**a**,**c**,**e**) are the corresponding optical images of gas sensors; the insert of **b** is its magnified image).

FE-SEM images, shown in Figure 2, present the structures and morphologies of three SnO_2_ samples. The optical images of the three fabricated gas sensors are displayed in the inset of Figure 2. Taken together, the three SnO_2_ samples show three-dimensional hierarchical structures assembled by one-dimensional nanorods. Figure 2a,b display FE-SEM images of the as-prepared SN-1 sample. It is obvious that the SN-1 sample consists of many nanorods and forms a novel radiating structure (urchin-like spheres). The diameter of this sphere is about 430 nm (Figure 2b). From the magnified FE-SEM image in the inset of Figure 2b, the diameter of these rods is approximately 20 nm. Additionally, each rod of SN-1 displays a sharp tip and smooth surface, and the rods are not closely stacked together but interlaced with one another in such a manner that results in urchin-like morphology, leaving sufficient space between these rods. When hydrothermal time in the preparation process is reduced to 12 h, the SN-2 sample was obtained. FE-SEM images of the SN-2 sample are shown in Figure 2c,d. The images indicate that the structure and morphology of the SN-2 sample has changed a little. Overall, the SN-2 sample is still a spherical flower-like structure assembled by many one-dimensional nanorods. However, the obvious difference is that the diameter of nanorods (55 nm) constructing the three-dimensional hierarchical structure is greater than SN-1, and the cross section of these nanorods is rectangular. It is more compact between nanorods, and these flower-like spheres are in bud and more similar to the seeding stage of the SN-1 sample. It can be inferred that prolonging hydrothermal reaction times promotes the continuous growth of the SN-2 sample. If the reaction time of the SN-2 sample is prolonged for 12 h, the SN-1 sample may be obtained. It should be noted that the above two samples are prepared when 0.04 g of citric acid was added, which plays a vital role in the formation of morphologies. The surface areas of the obtained samples are provided in Table 1. It can be observed that the SN-1 sample displays the highest surface area value among them.

**Figure 3:**
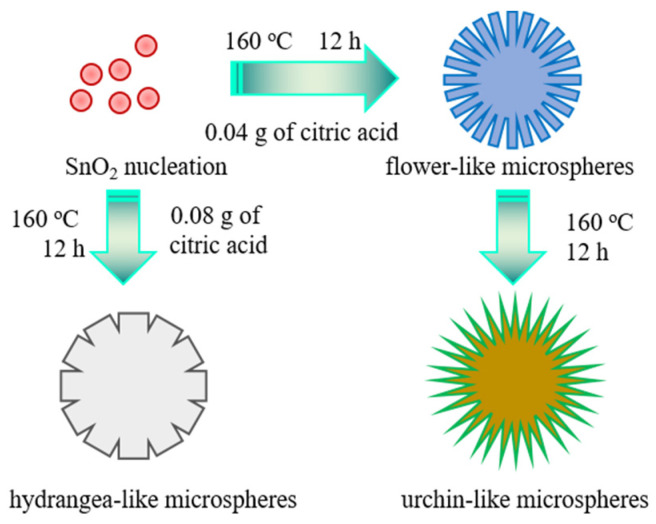
The possible growth mechanism.

In order to prove the important role of citric acid in the preparation process, another SN-3 sample was prepared by increasing the amount of citric acid to 0.08 g, while other conditions were consistent with those of the SN-2 sample. The FE-SEM images of the SN-3 sample are shown in Figure 2e,f. It can be observed that the SnO_2_ sample is still composed of one-dimensional nanorods, but the morphology and basic unit of the three-dimensional hierarchical structure have been changed. Compared with the SN-2 sample, the one-dimensional nanorod becomes thicker (145 nm), and it looks similar to hydrangeas. Many nanoparticles are distributed on the surface of nanorods, which results in a rough surface. These changes once again show that citric acid plays a significant role in the formation of sphere-like structures assembled by nanorods (Figure 3). Based on the above results and previous studies, the growth mechanism of the sphere-like SnO_2_ may be explained in the following equations.

(1) $$Sn^{4+}+4OH^{-}\to Sn{\left(OH\right)}_{4}$$

(2) $$Sn{\left(OH\right)}_{4}+2OH^{-}\to {\left[Sn{\left(OH\right)}_{6}\right]}^{2-}$$

(3) $${\left[Sn{\left(OH\right)}_{6}\right]}^{2-}\to SnO_{2}+2OH^{-}+2H_{2}O$$

Firstly, Sn(OH)_4_ is formed due to the reaction between Sn^4+^ and OH^−^ in the solution, as shown in Equation (1). Then, excess OH^−^ continues to react with the formed Sn(OH)_4_ to produce [Sn(OH)_6_]^2−^ (Equation (2)). However, with the progression of hydrothermal reactions, the generated [Sn(OH)_6_]^2−^ would be further dehydrated to form SnO_2_ (Equation (3)). In this manner, a SnO_2_ crystal nucleus would be formed. Meanwhile, the addition of citric acid has great impacts on continuous growth and final morphologies. It is well mentioned that such SnO_2_ morphology is related to the interaction of electrostatic interaction and van der Waals forces. Moreover, the formation of the rod structure is ascribed to the selective growth of SnO_2_ crystals on different planes. When citric acid is added into the solution, its anions form a complex chelate with Sn^4+^, which inhibits the nucleation of SnO_2_. [Sn(OH)_6_]^2−^ becomes a soft template due to weak interactions, inducing the crystal’s continual growth (001). Therefore, sphere-like SnO_2_ architectures assembled by rods are formed with the help of citric acid. However, the more detailed growth mechanism still needs further investigation.

### 3.2. Gas-Sensing Properties

**Figure 4:**
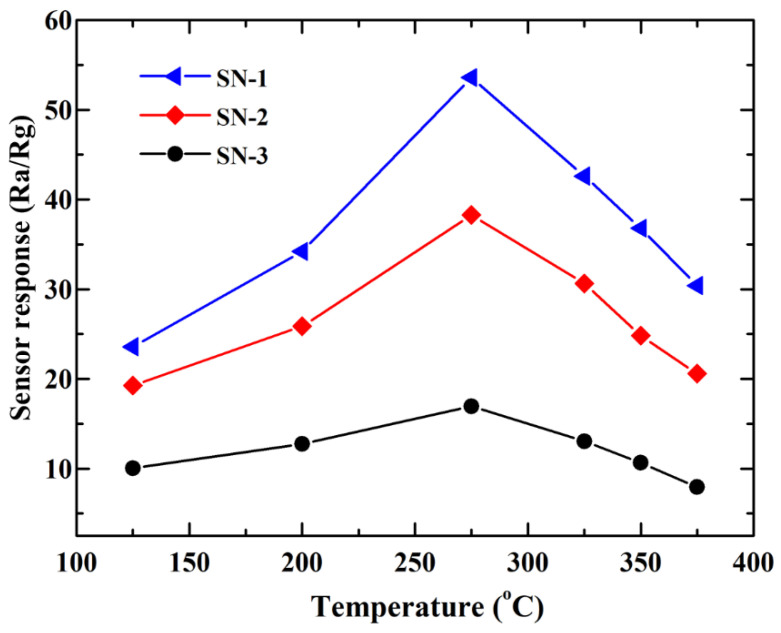
Sensor responses of as-prepared SnO_2_-based gas sensors towards 50 ppm HCHO vapor.

In order to investigate gas-sensing properties of the three hierarchical SnO_2_ microspheres relative to HCHO gas, gas-sensing measurements were carried out. It is well known that operating temperatures have important impacts on the gas-sensing behaviors of oxide-based gas sensors; thus, tests towards 50 ppm HCHO were firstly performed at different temperatures (125–375 °C). The sensor response of the three types of hierarchical SnO_2_-based gas sensors at various operating temperatures is displayed in Figure 4. From 125 to 275 °C, sensor responses of SN-1, SN-2, and SN-3-based gas sensors all continuously increased with an increase in operating temperature and achieved maximum values. However, when the sensor-operating temperature is beyond 275 °C, the further increase in operating temperature results in a decrease in sensor responses. As a result, it can be determined that the optimal operating temperature of the gas sensors is about 275 °C. Moreover, it can be observed that the SN-1-based gas sensor exhibits the highest sensor response value at all tested operating temperatures, followed by SN-2- and SN-3-based gas sensors. The maximum sensor responses of SN-1, SN-2, and SN-3 at 275 °C relative to 50 ppm HCHO gas molecular are 53.6, 38.3, and 17.0, respectively, which are summarized in Table 1.

**Figure 5:**
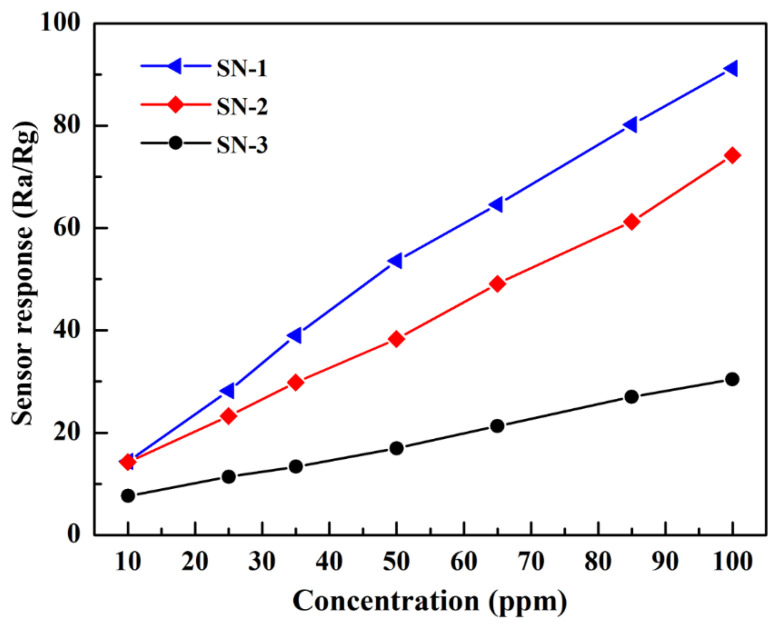
Sensor response curve of three SnO_2_-based gas sensors towards different HCHO concentrations at 275 °C.

The target gas concentration also strongly affects the gas-sensing properties of the gas sensor’s expected operating temperature. Thus, the sensor responses of sensors towards different gas concentrations were then measured at the optimal temperature of 275 °C, as shown in Figure 5. It can be clearly observed that with increasing concentrations of HCHO gas (10 ppm, 25 ppm, 35 ppm, 50 ppm, 65 ppm, 85 ppm, and 100 ppm), the sensor responses of the three SnO_2_-based gas sensors increase continuously. Among the three hierarchical SnO_2_ microspheres-based gas sensors, the sensor response of the SN-1-based gas sensor is always higher than the SN-2- and SN-3-based gas sensors. This result indicates the uniqueness of the structure of the SN-1 sample and realized gas-sensing properties relative to HCHO gas. 

**Figure 6:**
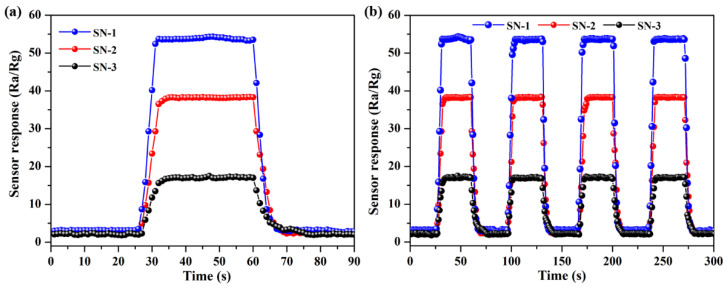
(**a**) Dynamic response and recovery curves of three SnO_2_ samples relative to 50 ppm HCHO at 275 °C. (**b**) The repeatability of three sensors towards 50 ppm HCHO at 275 °C.

Response and recovery behaviors are also vital parameters for gas sensors. Figure 6a shows dynamic response and recovery curves of the three SnO_2_ samples relative to 50 ppm HCHO at 275 °C. Moreover, basic unit morphology has a significant role on gas-sensing properties. Compared with SN-2 and SN-3, the SN-1 sample-based gas sensor displays higher sensor responses. Moreover, it is evident that sensor responses firstly increase and reach a maximum when the three SnO_2_-based gas sensors are exposed to 50 ppm HCHO vapor. The response and recovery times of the three sphere-like SnO_2_-based gas sensors are 5 s, 8 s, and 9 s; and 9 s, 11 s, and 15 s relative to 50 ppm HCHO at 275 °C, respectively. In addition, Figure 6b displays dynamic response and recovery curves for four cycles of three SnO_2_-based sensor responses. The slight change of sensor responses indicates good repeatability of the gas sensors.

**Figure 7:**
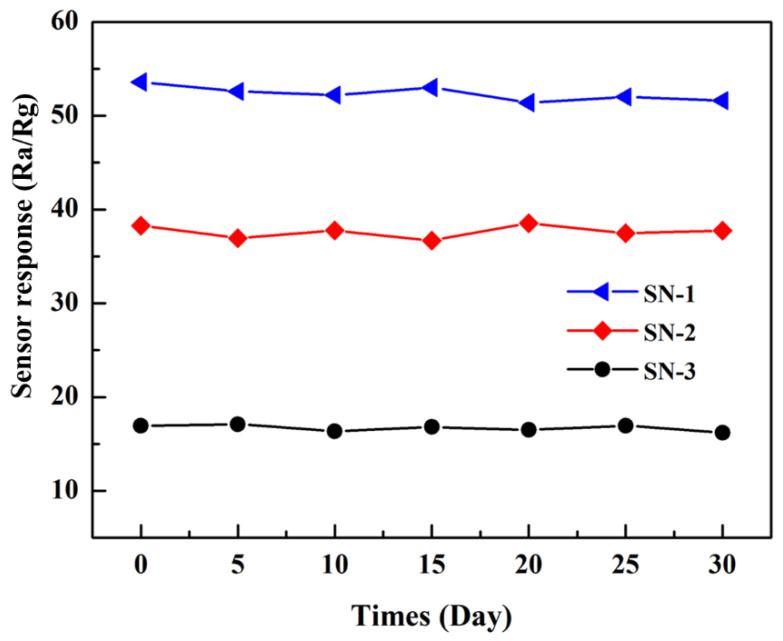
The long-term stability of three SnO_2_-based gas sensors towards 50 ppm HCHO at 275 °C.

In addition, the long-term stability of the three hierarchical SnO_2_ microspheres-based gas sensors towards 50 ppm HCHO vapor was tested for a month, and the results are shown in Figure 7. As exhibited in Figure 7, the three SnO_2_¬-based gas sensors display good long-term stability, and the SN-1-based sensor demonstrates better sensor response than that of SN-2- and SN-3-based gas sensors.

### 3.3. Gas-Sensing Mechanism

**Figure 8:**
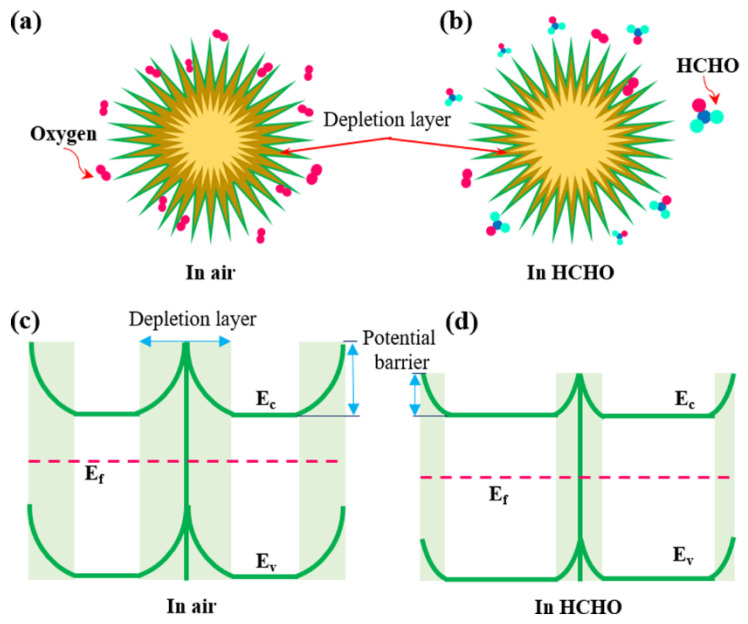
(**a**,**b**) Gas-sensing mechanism for SnO_2_-based HCHO sensor. (**c**,**d**) Schematic energy level diagram of SnO_2_ before and after exposure to HCHO.

As is known to all, the SnO_2_-based gas sensor is the surface-controlled sensor [25]. The gas-sensing mechanism could be elucidated by conductivity change, which is caused by surface interactions between the target gas and the SnO_2_-sensing surface (as shown in Figure 8). Firstly, when exposed to air, the oxygen in the air is adsorbed on the surface of the SnO_2_-sensing film to generate different types of oxygen ions (O_2_^−^, O^−^ and O^2−^) by trapping free electrons from the SnO_2_ conduction band. This results in a reduction in carrier concentration and can form an electron depletion layer on the surface of SnO_2_ nanomaterials. Consequently, the resistance of SnO_2_-based sensors in air atmospheres increases. Then, a surface interaction between HCHO gas and the oxygen ions occurs when the HCHO gas is introduced. This results in the release of electrons back to the SnO_2_ conduction band and forms carbon dioxide and water, which decreases the resistance of SnO_2_-based gas sensors. 

**Figure 9:**
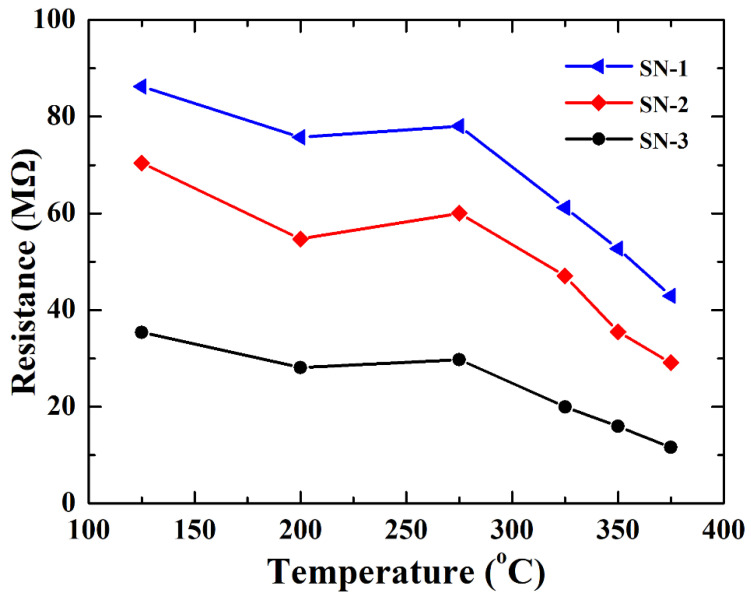
Working temperature dependence of resistance of SnO_2_-based gas sensor in air.

In order to further determine the effect of adsorbed oxygen on gas sensing, the resistance of the three gas sensors in air as a function of temperature was explored. As observed from Figure 9, the resistance gradually reduced from 125 to 375 °C. This result is attributed to two competitive processes. On the one hand, the conductance of three gas sensors increases with the rise of temperature due to intrinsic excitation. On the other hand, adsorbed oxygen traps electrons from the surface of the SnO_2_-sensing material, deteriorating the transport of electrons. However, when gas sensors are operated from 200 to 275 °C, resistance slightly increases, confirming that the amount of adsorbed oxygen obviously increases. However, when the temperature exceeds 275 °C, the resistance of all three sensors decreased, indicating that the intrinsic excitation is dominant at high working temperatures. This result is also consistent with the optimal working temperature in Figure 4.

Based on the aforementioned FE-SEM results, the three SnO_2_ samples are all composed of three-dimensional hierarchical sphere-like structures assembled by one-dimensional nanorods. However, the basic units of the three SnO_2_ samples are clearly different. It can be inferred that structural difference and surface area are the main reasons for the significant difference in gas-sensing performance. The nanorod diameter of SN-1 is the smallest, followed by SN-2 and SN-3. A smaller size offers a larger surface area, which corresponds to the surface area results provided in Table 1. Therefore, the SN-1 sample could provide more active adsorption sites for HCHO molecules. In addition, compared with SN-2 and SN-3, the SN-1 sample possesses more porous structure, offering more space for HCHO gas diffusion. Thus, the SN-1 sample-based gas sensor exhibits superior gas-sensing properties relative to HCHO gas than SN-2 and SN-3. A comparison between this study and other literature is also summarized in Table 1. Taken together, the as-prepared SnO_2_ hierarchical structures is a candidate for effective detection of HCHO.

## 4. Conclusions

In summary, three-dimensional hierarchical sphere-like SnO_2_ nanostructures with different basic units have been successfully prepared via a simple hydrothermal method. The results demonstrated that the diameter of one-dimensional nanorods as the basic unit is obviously different. The diameter of the nanorods can be changed by controlling hydrothermal time and the amount of citric acid. When comparing SN-2 and SN-3 samples, the SN-1 sample possesses smaller nanorods and better hierarchical structure, probably achieving different gas-sensing properties. Therefore, HCHO-sensing tests were then carried out. The sensor response values of SN-1, SN-2, and SN-3-based gas sensors towards 50 ppm HCHO vapor at 275 °C are 53.6, 38.3, and 17.0 at 275 °C, respectively. The response and recovery times are 5 s, 8 s, and 9 s; and 9 s, 11 s, and 15 s, respectively. This test result indicates that the SN-1-based gas sensor displays the best sensor response properties among the three SnO_2_-based gas sensors at the same operating temperature, which is ascribed to the fact that the SN-1 sample posesses more active adsorption sites and diffusion spaces.

## Figures and Tables

**Table 1:** Comparison of different SnO_2_ structures in this study and other literature.

Material	Surface Area (m^2^/g)	Working Temperature (°C)	Gas Concentration (ppm)	Sensor Response towards HCHO	Ref.
SN-1	29.367	275	50	53.6	This work
SN-2	24.543	275	50	38.3	
SN-3	13.446	275	50	17.0	
petal-like SnO_2_	/	180	100	12.1	[22]
Bi-doped SnO_2_ flowers		170	100	36.2	
SnO_2_ microspheres		200	100	38.3	[23]
Sb-doped SnO_2_ nanoflowers		280	100	45	[24]

## Data Availability

Data can be available upon request from the authors.
